# Association of High-Potency Cannabis Use With Mental Health and Substance Use in Adolescence

**DOI:** 10.1001/jamapsychiatry.2020.1035

**Published:** 2020-05-27

**Authors:** Lindsey A. Hines, Tom P. Freeman, Suzanne H. Gage, Stanley Zammit, Matthew Hickman, Mary Cannon, Marcus Munafo, John MacLeod, Jon Heron

**Affiliations:** 1 Population Health Science, Bristol Medical School, University of Bristol, Bristol, United Kingdom; 2 Addiction and Mental Health Group, Department of Psychology, University of Bath, Bath, United Kingdom; 3 Institute of Psychiatry, Psychology & Neuroscience, National Addiction Centre, London, United Kingdom; 4 Clinical Psychopharmacology Unit, University College London, London, United Kingdom; 5 Addiction Group, Department of Psychological Sciences, University of Liverpool, Liverpool, United Kingdom; 6 Medical Research Council Centre for Neuropsychiatric Genetics and Genomics, Cardiff University, Cardiff, United Kingdom; 7 Department of Psychiatry, Royal College of Surgeons in Ireland, Dublin, Ireland; 8 Experimental Psychology, University of Bristol School of Psychological Science, Bristol, United Kingdom

## Abstract

**Importance:**

Cannabis use is consistently linked to poorer mental health outcomes, and there is evidence that use of higher-potency cannabis increases these risks. To date, no studies have described the association between cannabis potency and concurrent mental health in a general population sample or addressed confounding using longitudinal data.

**Objective:**

To explore the association between cannabis potency and substance use and mental health outcomes, accounting for preceding mental health and frequency of cannabis use.

**Design, Setting, and Participants:**

This cohort study used data from the Avon Longitudinal Study of Parents and Children, a UK birth cohort of participants born between April 1, 1991, and December 31, 1992. Present data on outcomes and exposures were collected between June 2015 and October 2017 from 1087 participants at 24 years of age who reported recent cannabis use.

**Exposures:**

Self-reported type of cannabis most commonly used in the past year, coded to a binary exposure of use of high-potency cannabis or lower-potency cannabis.

**Main Outcomes and Measures:**

Outcomes were reported frequency of cannabis use, reported cannabis use problems, recent use of other illicit drugs, tobacco dependence, alcohol use disorder, depression, generalized anxiety disorder, and psychotic-like experiences. The study used secondary data; consequently, the hypotheses were formulated after data collection.

**Results:**

Past-year cannabis use was reported by 1087 participants (580 women; mean [SD] age at onset of cannabis use, 16.7 [3.0] years). Of these, 141 participants (13.0%) reported the use of high-potency cannabis. Use of high-potency cannabis was associated with increased frequency of cannabis use (adjusted odds ratio [AOR], 4.38; 95% CI, 2.89-6.63), cannabis problems (AOR, 4.08; 95% CI, 1.41-11.81), and increased likelihood of anxiety disorder (AOR, 1.92; 95% CI, 1.11-3.32). Adjustment for frequency of cannabis use attenuated the association with psychotic experiences (AOR 1.29; 95% CI, 0.67-2.50), tobacco dependence (AOR, 1.42; 95% CI, 0.89-2.27), and other illicit drug use (AOR, 1.29; 95% CI, 0.77-2.17). There was no evidence of association between the use of high-potency cannabis and alcohol use disorder or depression.

**Conclusions and Relevance:**

To our knowledge, this study provides the first general population evidence suggesting that the use of high-potency cannabis is associated with mental health and addiction. Limiting the availability of high-potency cannabis may be associated with a reduction in the number of individuals who develop cannabis use disorders, the prevention of cannabis use from escalating to a regular behavior, and a reduction in the risk of mental health disorders.

## Key Points

**Question:**

Does use of high-potency cannabis (compared with use of low-potency cannabis) increase risks for problems resulting from cannabis use, common mental disorders, and psychotic experiences after controlling for early-life mental health symptoms and frequency of use?

**Findings:**

In this cohort study of 1087 participants who reported cannabis use in the previous year, after adjusting for frequency of cannabis use and early adolescent mental health, use of high-potency cannabis was associated with a significant increase in the frequency of cannabis use, likelihood of cannabis problems, and likelihood of anxiety disorder. Those using high-potency cannabis had a small increase in the likelihood of psychotic experiences; however, this risk was attenuated after adjustment for frequency of cannabis use.

**Meaning:**

Risks for cannabis use problems and anxiety disorders are higher among those reporting use of high-potency cannabis; provision of public health messaging regarding the importance of reducing both frequency of cannabis use and the potency of the drug, as well as limiting the availability of high-potency cannabis, may be effective for reducing these risks.

## Teaser

This cohort study examines the association between cannabis potency and substance use and mental health outcomes, accounting for preceding mental health and frequency of cannabis use.

## Introduction

Globally, cannabis is the most commonly used internationally regulated drug,^1^ and policy on its use is becoming more liberal worldwide.^2^ The primary psychoactive component of cannabis is Δ^9^-tetrahydrocannabinol (THC). The potency (concentration of THC) may be an important factor in the association between cannabis use and mental health. Experimental studies indicate that THC intoxication is dose dependent, with higher doses causing greater memory impairment and transient psychotic-like symptoms.^3^ Policy liberalization has been accompanied by proliferation of high-potency cannabis in legal markets,^4,5^ and THC concentrations have increased in markets where cannabis remains illegal.^6^

Cannabis use is consistently linked to poorer mental health outcomes,^7,8^ and there is evidence that higher-potency cannabis is associated with higher risks. A case-control study of first-episode psychosis in England found that those who self-reported using higher-potency cannabis were twice as likely to have a psychotic disorder, compared with participants who did not use cannabis.^9^ When the study was replicated in a multinational case-control study of first-episode psychosis across 11 sites in Europe and Brazil, the incidence of psychosis across sites was positively correlated with the prevalence of high-potency cannabis use in the site-specific control samples.^10^ In a self-selecting sample of people who use internationally regulated drugs, the use of high-potency strains of cannabis was associated with self-report of lifetime depression^11^ and cannabis dependence.^12^ These findings indicate that the availability of high-potency cannabis may increase risks of poorer mental health, addiction, and need for treatment among those who are using the drug.

The strength of association between the use of cannabis and mental health outcomes is increased when cannabis use is frequent; consequently, increased frequency of use may confound the association between cannabis potency and mental health outcomes.^12^ Understanding the extent to which harms of high-potency cannabis are due to the THC content of the drug, and the extent to which these harms may be accounted for by increased frequency of use, is important for informing policy decisions around taxation and limits on drug potency.

To our knowledge, to date, no studies of the association between cannabis potency and mental health have been conducted in a general population sample. General population studies can provide a valid estimate of the association between mental health outcomes and cannabis potency at the population level, which may be crucial for informing policy makers and clinical service providers. We use data from the Avon Longitudinal Study of Parents and Children (ALSPAC), a large general population birth cohort where contemporaneous data were collected when participants were 24 years of age on cannabis potency, cannabis use frequency, and validated measures of mental health outcomes, and prospective measures of adolescent mental health were collected from participants up to 24 years of age.

The aims of our study were to (1) describe the use of different potencies of cannabis among a population of UK adolescents; (2) explore the association between cannabis potency and problems resulting from cannabis use, use and disordered use of other substances, common mental disorders, and psychotic experiences (PEs) by comparing those who use high-potency cannabis with those who use lower potency cannabis; and (3) determine the extent to which such associations are explained by adolescent mental health at 12 to 13 years of age, age at onset of cannabis use, and current frequency of cannabis use.

## Methods

### Study Population

**Figure 1.**
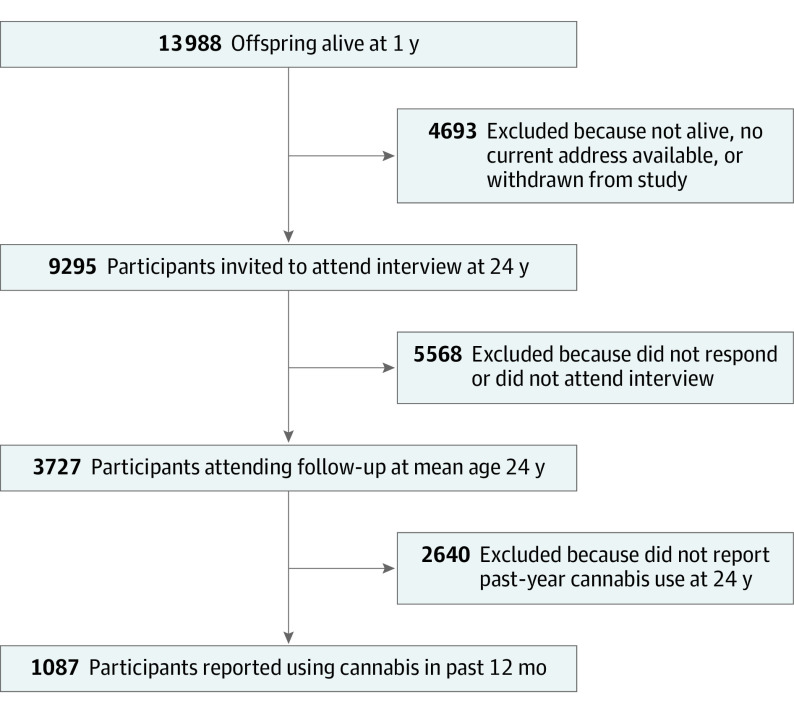
Flow Diagram Showing the Avon Longitudinal Study of Parents and Children Analysis Sample

ALSPAC is a UK population-based birth cohort, the methods of which have previously been outlined^13,14^ (see eAppendix 1 in the Supplement for full details). The sample for the present analyses comprised the 1087 participants who reported on their past-year cannabis use while attending the ALSPAC clinic between June 2015 and October 2017 at a mean (SD) age of 24.0 (0.8) years (Figure 1). Data on the 2085 individuals who participated in the assessment at 24 years of age but were excluded based on reporting no recent cannabis use are available in eTable 3 in the Supplement (see also eAppendix 1 in the Supplement for further details on all the measures described). Ethical approval for the study was obtained from the ALSPAC Ethics and Law Committee and the local Research Ethics Committees. Written informed consent for the use of data collected via questionnaires and clinics was obtained from participants following the recommendations of the ALSPAC Ethics and Law Committee at the time.

### Exposure

Those who reported past-year cannabis use were asked “What type of cannabis have you most commonly used or taken in the last 12 months?” and were able to select from the following options: “herbal cannabis/marijuana,” “skunk/other stronger types of herbal cannabis,” “hashish/resin/solid,” “other,” or “don’t know.” Consistent with previous research that has validated self-reported data on 3 cannabis types against quantified concentrations of THC and cannabidiol among young UK cannabis users,^15^ we categorized cannabis as either high potency (typically ≥10% THC; “skunk/other stronger types of herbal cannabis”) or low potency (typically <10% THC; “herbal cannabis/marijuana,” “hashish/resin/solid,” or “other”).^10^

### Outcomes

#### Cannabis Use Frequency at 24 Years of Age

Participants were asked “in the last 12 months, how often have you used cannabis?” This variable was dichotomized to “Monthly use or less” or “weekly/daily use.”

#### Problematic Cannabis Use at 24 Years of Age

Those who self-reported 2 or more items on the Cannabis Abuse Screening Test^16^ within the past year were classified as having recently experienced problems associated with their cannabis use.

#### Other Substance Use and Dependencies at 24 Years of Age

Participant self-report of the use of any illicit drugs in the past 12 months was categorized as recent other illicit drug use. The reference group was composed of those who reported no recent use of these other illicit drugs (including those who had never used these drugs). Participants were categorized as nicotine dependent if they self-reported daily nicotine use and reported 3 or more criteria on the Fagerström Test for Nicotine Dependence,^17^ indicating low, medium, high, or very high nicotine dependence. The reference group was composed of those who met criteria for very low dependence and those who were not using nicotine every day or had never smoked. The *DSM-5* criteria for alcohol use disorder (AUD)^18^ were used to identify participants experiencing alcohol use problems. Participants were categorized as experiencing moderate or severe AUD if they self-reported 4 or more of the AUD criteria. The reference group was composed of those who reported less than 4 of the AUD criteria (including individuals who had never drunk alcohol).

#### Mental Health at 24 Years of Age

Participants completed a self-administered computerized Clinical Interview Schedule–revised,^19^ a tool for lay interviewers to assess psychiatric disorders in the community. Participants who met the criteria for moderate or severe *DSM-IV*^20^ major depressive disorder at time of the interview were categorized as experiencing depression. Participants who met *DSM-IV* criteria for generalized anxiety disorder^20^ were categorized as experiencing anxiety. Participants were rated on PEs using the PLIKS (psychosis-like symptoms) semistructured interview administered by trained researchers.^21,22^ Those who were rated as having suspected or definite hallucinations, delusions, or thought interference in the past 12 months, which were either frequent (at least monthly) or caused them distress (reported as quite distressing or very distressing), were classified as having had a recent PE. For the purposes of these analyses, PEs were excluded if they happened only when the participant was either falling asleep or waking up, was ill with a high temperature, or within 2 hours of drinking alcohol or taking drugs.

### Covariates

#### Prospective Measures From Early Childhood and Adolescence

Childhood socioeconomic position was assessed through measures from maternal questionnaires completed during pregnancy; variables were maternal educational attainment and parents’ occupation class. A child’s racial/ethnic background was derived from parents’ reported race/ethnicity (coded as white or black and minority ethnic group).

To account for mental health symptoms preceding the onset of cannabis use (mean age at onset of cannabis use in sample, 16.7 years; 95% CI, 16.5-16.9 years), a continuous score measure of depression symptoms at 13 years of age was included in analyses of major depressive disorder and generalized anxiety disorder. These symptoms were assessed through the self-completed Mood and Feelings Questionnaire at 13 years of age,^23^ a tool for measuring depression in children and young people. The number of PEs (assessed by PLIKS^21^) reported at 12 years of age was included in the analysis of the outcome of PEs at 24 years of age.

Age at onset of cannabis use was self-reported in ALSPAC at 14, 15, 16, 18, 20, 22, and 24 years of age. Participants who reported lifetime use of cannabis at any of these points were asked at what age they first used cannabis. Participants’ earliest report of age at first cannabis use was used to derive a variable of age at onset of cannabis use.

### Statistical Analysis

All analyses were conducted in Stata, version 15.1 (Stata Corp). The association between use of high-potency cannabis and substance use and mental health at 24 years of age was analyzed using univariable and multivariable logistic regression, with cannabis potency as the independent variable. Three separate multivariable models were performed for each outcome: (1) adjusting for sex and childhood socioeconomic position; (2) additional adjustment for age at onset of cannabis use for models of substance use outcomes, depression symptom score at 13 years of age for models of major depressive disorder and generalized anxiety disorder outcomes, and PEs at 12 years of age for the model with PE as the outcome (time points selected to ensure mental health symptoms preceded onset of cannabis use); and (3) adjusted as in model 2, with inclusion of a categorical measure of cannabis use frequency. This measure allowed estimation of the extent to which preexisting symptoms of mental health disorders and the frequency of cannabis use explained any association between the use of high-potency cannabis and substance use and mental health outcomes. Results are presented as odds ratios (ORs) with 95% CIs. Propensity score models were applied to complete case data as a sensitivity test (eAppendix 2 and eTables 1 and 2 in the Supplement).

### Missing Data and Imputation

As outcomes and exposures were collected at the same point, most missing data were in the covariates assessed at earlier ages (eAppendix 1 and eTable 3 in the Supplement). Missing data in all analysis variables (exposures, outcomes, and covariates) were addressed through multiple imputation using chained equations, which uses a series of univariate regression models to impute each incomplete variable sequentially. Each model included all other analysis variables, along with the following auxiliary variables: race/ethnicity, experiencing bullying up to 16 years of age, parental separation up to 16 years of age, parental mental health problems up to 16 years of age, parental substance use up to 16 years of age, Mood and Feelings Questionnaire score at 16 and 18 years of age, number of self-reported psychotic-like experiences at 14 years of age, and conduct disorder symptoms up to 13 years of age. Estimates were obtained by pooling results across 40 imputed data sets using the Rubin rules, and assessment of Monte Carlo variability confirmed this as a suitable number of imputations.^24^

## Results

Of the 1087 participants reporting past-year cannabis use at 24 years of age, 12.8% (number estimated from imputed proportions) reported the use of high-potency cannabis (Table 1). Use of lower-potency forms of cannabis was reported by 87.2% (number estimated from imputed proportions) of those who used cannabis in the past year (see eTable 3 in the Supplement for data on this and on all analysis variables). Use of high-potency cannabis was more common than use of lower-potency cannabis in those who were male (71.6% vs 43.3%) and those who reported regular cannabis use (56.8% vs 17.6%), recent cannabis use problems (10.1% vs 0.8%), recent use of other illicit drugs (82.9% vs 66.5%), tobacco dependence (37.0% vs 15.1%), AUD (15.1% vs 10.0%), depression (11.7% vs 9.7%), generalized anxiety disorder (19.1% vs 11.6%), and PE (12.4% vs 7.1%) (Table 1).

**Table 1.** Association Between Type of Cannabis and Demographic, Substance Abuse, and Mental Health Outcomes in 1087 Participants Who Reported Recent Cannabis Use

Characteristic	Cannabis use, %^a^		*P* value^b^
	High potency (n = 141)	Lower potency (n = 946)	
Regular cannabis use	56.8	17.6	≤.001
Cannabis use problems	10.1	0.8	≤.001
Use of other illicit drugs	82.9	66.5	≤.001
Tobacco dependence	37.0	15.1	≤.001
Alcohol use disorder	15.1	10.0	≤.001
Major depression (moderate or severe symptoms)	11.7	9.7	≤.001
Generalized anxiety disorder	19.1	11.6	≤.001
Psychotic-like experiences	12.4	7.1	≤.001
Male sex	71.6	43.4	≤.001
Low maternal educational level	19.2	13.1	≤.001
Lower parental occupational class	32.2	29.2	≤.001
Black or minority ethnic group	5.3	5.3	.94
Age at onset of cannabis use, mean (95% CI), y	14.7 (14.3-15.1)	16.9 (16.8-17.2)	NA
MFQ score at 13 y, mean (95% CI)	5.6 (4.65-6.49)	5.6 (5.26-5.95)	NA
No. PEs at 12 y, mean (95% CI)	0.33 (0.18-0.48)	0.20 (0.16-0.24)	NA

Abbreviations: MFQ, Mood and Feelings Questionnaire; NA, not applicable; PEs, psychotic experiences.

^a^ All numbers estimated from imputed proportions.

^b^ Determined by χ^2^ test.

### Cannabis Use Outcomes

**Figure 2.**
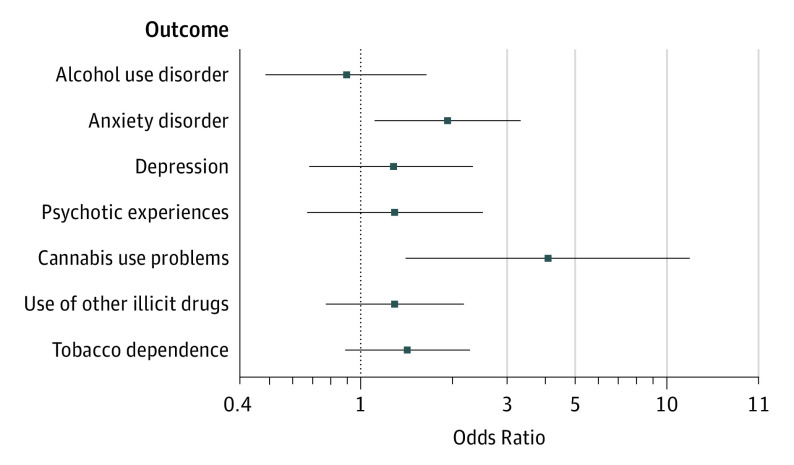
Fully Adjusted Associations Between the Use of High-Potency Cannabis and Outcomes Including fully adjusted associations for sociodemographics, longitudinal mental health, and frequency of use (see Methods). The horizontal bars indicate 95% CIs, and the dotted vertical line indicates the comparator group (individuals who reported use of lower-potency cannabis).

There was an unadjusted association between the use of high-potency cannabis and frequency of cannabis use at 24 years of age (OR, 6.21; 95% CI, 4.24-9.11) (Table 2). This association was attenuated by adjustment for sociodemographic factors and by the age at onset of cannabis use, but those reporting use of high-potency cannabis remained more than 4 times as likely to report using cannabis at least weekly compared with those reporting use of lower-potency forms of cannabis (adjusted OR [AOR], 4.38; 95% CI, 2.89-6.63). There was also an unadjusted association between the use of high-potency cannabis and reporting recent cannabis problems (OR, 13.17; 95% CI, 5.41-32.04). This association was attenuated by adjustment for age at onset of cannabis use and frequency of cannabis use, but people reporting the use of high-potency cannabis were still more than 4 times as likely to report having recently experienced problems associated with their cannabis use (AOR, 4.08; 95% CI, 1.41-11.81) (Table 2 and Figure 2).

**Table 2.** Logistic Regression Analysis of the Association Between High-Potency Cannabis and Substance Use and Mental Health Outcomes^a^

Outcome variable	Univariable OR (95% CI)	*P* value	Adjusted for childhood sociodemographic factors, AOR (95% CI)	*P* value	Adjusted for prospective mental health measures, AOR (95% CI)	*P* value	Adjusted for frequency of cannabis use, AOR (95% CI)	*P* value
Regular cannabis use	6.21 (4.24-9.11)	≤.001	5.81 (3.90-8.65)	≤.001	4.38 (2.89-6.63)^b^	≤.001	NA	NA
Recent cannabis use problems	13.17 (5.41-32.04)	≤.001	13.52 (5.28-34.60)	≤.001	8.45 (3.04-23.50)^b^	≤.001	4.08 (1.41-11.81)	.009
Recent use of other illicit drugs	2.47 (1.53-3.97)	≤.001	2.19 (1.35-3.56)	.002	1.50 (0.91-2.49)^b^	.11	1.29 (0.77-2.17)	.34
Tobacco dependence	3.31 (2.23-4.92)	≤.001	3.30 (2.18-4.99)	≤.001	2.05 (1.31-3.19)^b^	.002	1.42 (0.89-2.27)	.14
Alcohol use disorder	1.60 (0.94-2.73)	.08	1.49 (0.86-2.56)	.15	0.99 (0.56-1.76)^b^	.97	0.90 (0.49-1.64)	.73
Major depression (moderate or severe symptoms)	1.24 (0.70-2.18)	.46	1.61 (0.89-2.93)	.12	1.54 (0.84-2.82)^c^	.16	1.28 (0.68-2.32)	.44
Generalized anxiety disorder	1.77 (1.09-2.86)	.02	2.35 (1.41-3.92)	≤.001	2.28 (1.36-3.83)^c^	.002	1.92 (1.11-3.32)	.02
Psychotic-like experiences	1.81 (1.01-3.24)	.047	2.03 (1.10-3.73)	.02	1.86 (1.00-3.46)^d^	.05	1.29 (0.67-2.50)	.45

Abbreviations: AOR, adjusted odds ratio; NA, not applicable; OR, odds ratio.

^a^ All results estimated from imputed data. Multivariable model adjustment is incremental.

^b^ Age at onset of cannabis use.

^c^ Depression symptom score at 13 years of age.

^d^ Number of psychotic experiences at 12 years of age.

### Substance Use Outcomes

Those who reported the use of high-potency cannabis were more than twice as likely to report recent use of other illicit drugs within the past 12 months (OR, 2.47; 95% CI, 1.53-3.97) and more than 3 times as likely to report tobacco dependence (OR, 3.31; 95% CI, 2.23-4.92) (Table 2). These associations were largely attenuated by adjustment for sociodemographic factors, age at cannabis onset, and frequency of cannabis use (recent use of other illicit drugs: AOR, 1.29; 95% CI, 0.77-2.17; and tobacco dependence: AOR, 1.42; 95% CI, 0.89-2.27). There was little evidence of an elevation in the likelihood of AUD among participants who reported use of high-potency cannabis after adjustment for sociodemographic variables, age at onset of cannabis use, and frequency of cannabis use (AOR, 0.90; 95% CI, 0.49-1.64) (Table 2 and Figure 2).

### Mental Health Outcomes

Depression was slightly more common in the high-potency cannabis group, but there was little statistical evidence that high potency was associated with depression (AOR, 1.28; 95% CI, 0.68-2.34) (Table 2). There was evidence that use of high-potency cannabis was associated with a moderate elevation in likelihood of generalized anxiety disorder (OR, 1.77; 95% CI, 1.09-2.86). The strength of this association was increased slightly after adjustment for sociodemographic variables, depression symptoms at 14 years of age, and frequency of cannabis use (AOR, 1.92; 95% CI, 1.11-3.32).

Participants reporting the use of high-potency cannabis were almost twice as likely to report frequent or distressing PEs (OR, 1.81; 95% CI, 1.01-3.24) (not occurring directly after drug use) (Table 2). However, evidence of association was weakened after adjustment for frequency of cannabis use (AOR, 1.29; 95% CI, 0.67-2.50) (Table 2 and Figure 2).

## Discussion

The present study restricts analyses to individuals who have used different forms of cannabis to inform understanding of the implications of the proliferation of high-potency cannabis in legal markets.^4,5^ In a general population sample of young people in the UK, individuals who use high-potency cannabis (compared with those using lower-potency forms of cannabis) are more likely to be using cannabis regularly, more likely to report having recently experienced problems associated with their cannabis use, and more likely to concurrently be experiencing use of other illicit drugs, tobacco dependence, AUD, generalized anxiety disorder, and PEs. After adjustment for age at onset of cannabis use or for early adolescent measures of psychopathologic conditions and frequency of cannabis use, high-potency cannabis was associated with increases in the likelihood of frequent cannabis use, having recently experienced problems associated with cannabis use, and the likelihood of experiencing generalized anxiety disorder. The results provide a profile of individuals who use high-potency cannabis, indicating that this behavior is more common among individuals who are male, grow up in families with a low socioeconomic status, experience early PEs, and report early-onset cannabis use.

The outcomes regarding substance use and mental health may reflect shared predisposing risk factors that could also lead people to select the most potent drug available.^25^ As the sample excluded those who had not used cannabis, increases in risk for mental health outcomes associated with the use of high-potency cannabis are unlikely to be conflated by a shared liability to drug use and mental health disorders. However, evidence shows that shared risk factors underlie both exposure to cannabis and progression to development of dependence.^26^ The profile of substance use outcomes and early-life experiences among those who use high-potency cannabis in the present study indicates that there may be overlapping risk factors between development of substance use and mental health disorders and the selection of higher-potency forms of cannabis. We have sought to adjust for these factors, but further consideration of the role of cannabis potency in the causal path to mental health disorders is warranted.

The estimate in the present study for the likelihood of depression among those using high-potency cannabis is similar to estimates observed in a previous study of a self-selecting sample of drug-using participants (OR, 1.18; 95% CI, 1.11-1.25).^11^ However, the estimate in the present study demonstrating an increase in the likelihood of generalized anxiety disorder among those using high-potency cannabis is in contrast to the negligible increase in likelihood of lifetime anxiety disorder observed in a previous study (OR, 1.05; 95% CI, 0.98-1.12).^11^ This discrepancy may be because the previous study relied on self-reported lifetime diagnosis,^11^ whereas the present study used *DSM-IV*–validated measures of depression and generalized anxiety disorder at the time of assessment. A recent systematic review has indicated that cannabis use in adolescence is associated with an increased likelihood of lifetime depression and anxiety,^8^ although few studies had considered the potency or frequency of cannabis use.

A better understanding of the association between potency and frequency of cannabis use is required to gain a clearer understanding of the independent association of potency with mental health. We found similar effect sizes for the association between the use of high-potency cannabis and report of frequent or distressing PEs (not associated with drug use) as those observed in case-control studies of first-episode psychosis.^9,10^ However, in our study, we observed a substantial attenuation in effect size, by approximately 66%, after adjustment for frequency of cannabis use. The present findings on cannabis problems and frequency of use are consistent with previous evidence for a positive association between days of use of high-potency cannabis and severity of dependence.^12^ In this previous study there was a linear association between number of days of high-potency cannabis use per month and severity of dependence score (β = 0.15; 95% CI, 0.02-0.28). Increased frequency of cannabis use could plausibly cause individuals to use higher-potency cannabis through the development of tolerance to the effects of cannabis; if so, then it is appropriate to consider cannabis use frequency as a confounding factor in the association with mental health. However, if the use of higher-potency cannabis leads to increased frequency of use, plausibly through high-potency cannabis delivering THC more efficiently than lower-potency cannabis, it would be more appropriate to explore the mediating effect of cannabis frequency.

### Strength and Limitations

This study has some strengths. To our knowledge, this study is the first to describe the association between cannabis potency and concurrent mental health and substance abuse in a general population sample, and the first to use longitudinal data to address confounding by early mental health symptoms and age at onset of cannabis use in this association.

This study also has some limitations. First, given that the data were collected in the context of an illegal cannabis market, we cannot be certain participants are accurately informed about the potency of the cannabis they are using. It is plausible that the ability to identify type of cannabis is higher among those frequently using the drug, although evidence suggests that frequency of use does not moderate the association between self-reported identification of cannabis type and actual THC concentration in young UK cannabis users.^15^

Second, the exposures and outcomes that we examined were cross-sectional. Because questions on the type of cannabis used were only asked at 24 years of age, it is plausible that the presence of anxiety disorder or PEs have led to the use of high-potency cannabis at 24 years of age. Adjusting for measures of psychopathologic conditions in adolescence had little effect on our estimates.

Third, recent use of high-potency cannabis was reported by only 12.8% of participants, which may result in some of the analyses being underpowered (eTable 3 in the Supplement). Fourth, as a result of attrition within ALSPAC, those who took part in the wave of the study that took place at 24 years of age were more likely to be white, female, and more affluent than the population from which the participants were originally drawn.^14^ In addition, because there is a dearth of nationally representative data on the demographics of those who use cannabis, we cannot be certain the present sample is representative of the population of cannabis users in the UK or globally. The analyses would benefit from replication in larger, representative samples, but to our knowledge, ALSPAC is the only longitudinal general population sample to include measures of cannabis potency. Characteristics of those who did not report cannabis use at 24 years of age are in eTable 4 in the Supplement.

## Conclusions

The use of high-potency cannabis may occur as part of a profile of other illicit drug use and substance dependency, likely owing to shared risk factors underlying these behaviors and the selection of high-potency strains. The present study suggests that risks for cannabis use problems and anxiety disorders are further increased among those reporting the use of high-potency cannabis, even after accounting for sociodemographic factors, adolescent mental health, and frequency of cannabis use. Providing public health messaging regarding the importance of reducing both the frequency of cannabis use and the potency of the drug, as well as limiting the availability of high-potency cannabis, may be effective for reducing the harms associated with cannabis use.

## Notes

Supplement. **eAppendix 1.** Methods **eAppendix 2.** Sensitivity Test Using Propensity Score Models in Complete Case Data **eTable 1.** Variables and Interactions Included in Propensity Score **eTable 2.** Effect Sizes for Models of Relationship Between Cannabis Potency and Study Outcomes, Comparing Multivariable Adjustment and Inverse Probability Weighting by Propensity Score **eTable 3.** Sample Characteristics of 1087 Participants Who Reported Recent Cannabis Use (Numbers and Proportions From Complete-Case Data) **eTable 4.** Prevalence of Demographic, Substance Abuse and Mental Health Outcomes in 2805 Participants Who Did Not Report Recent Cannabis Use (Numbers and Proportions From Complete-Case Data) **eReferences.**
