# Supplementary material for: Association of High-Potency Cannabis Use With Mental Health and Substance Use in Adolescence
Source: JAMA Psychiatry. 2020 May 27;77(10):1044–51. doi: 10.1001/jamapsychiatry.2020.1035 (PMC7254445; doi:10.1001/jamapsychiatry.2020.1035)
Supplement: Supplement. — eAppendix 1. Methods eAppendix 2. Sensitivity Test Using Propensity Score Models in Complete Case Data eTable 1. Variables and Interactions Included in Propensity Score eTable 2. Effect Sizes for Models of Relationship Between Cannabis Potency and Study Outcomes, Comparing Multivariable Adjustment and Inverse Probability Weighting by Propensity Score eTable 3. Sample Characteristics of 1087 Participants Who Reported Recent Cannabis Use (Numbers and Proportions From Complete-Case Data) eTable 4. Prevalence of Demographic, Substance Abuse and Mental Health Outcomes in 2805 Participants Who Did Not Report Recent Cannabis Use (Numbers and Proportions From Complete-Case Data) eReferences. [file jamapsychiatry-e201035-s001.pdf]

Supplementary Online Content

Hines LA, Freeman TP, Gage SH, et al. Association of high-potency cannabis use with mental health and substance use in adolescence. *JAMA Psychiatry.* Published online May 27, 2020. doi:10.1001/jamapsychiatry.2020.1035

eAppendix 1. Methods
eAppendix 2. Sensitivity Test Using Propensity Score Models in Complete Case Data
eTable 1. Variables and Interactions Included in Propensity Score
eTable 2. Effect Sizes for Models of Relationship Between Cannabis Potency and Study Outcomes, Comparing Multivariable Adjustment and Inverse Probability Weighting by Propensity Score
eTable 3. Sample Characteristics of 1087 Participants Who Reported Recent Cannabis Use (Numbers and Proportions From Complete-Case Data)
eTable 4. Prevalence of Demographic, Substance Abuse and Mental Health Outcomes in 2805 Participants Who Did Not Report Recent Cannabis Use (Numbers and Proportions From Complete-Case Data)
eReferences.

This supplementary material has been provided by the authors to give readers additional information about their work.

© 2020 Hines LA et al. *JAMA Psychiatry*.

eAppendix 1. Methods

Study population

Pregnant women in the former Avon Health Authority in south-west England who had an estimated date of delivery between 1 April 1991 and 31 December 1992 were invited to take part, resulting in a cohort of 14 541 pregnancies and 13,988 children alive at 1 year of age. When the oldest children were approximately 7 years of age, an attempt was made to bolster the initial sample with eligible cases who had failed to join the study originally. The total sample size for analyses using any data collected after the age of seven is therefore 15,454 pregnancies, resulting in 15,589 foetuses. Of these 14,901 were alive at 1 year of age.[1] Ethical approval for this study was obtained from the ALSPAC Law and Ethics Committee and the Local Research Ethics Committees. The ALSPAC study website contains details of all the data available through a fully searchable data dictionary (http://www.bristol.ac.uk/alspac/researchers/our-data/). Study data were collected and managed using REDCap electronic data capture tools hosted at University of Bristol.[2,3]

Measures

Outcomes

Cannabis use frequency at age 24

Participants were asked "in the last 12 months, how often have you used cannabis?" with the options "not in the last 12 months", "once or twice", less than monthly", "monthly", "weekly", "daily or almost daily".

Problematic cannabis use at age 24

Those who endorsed two or more of the following Cannabis Abuse Screening Test (CAST)[6] items within the past year were classified as having recently experienced problems as a result of their cannabis use: using cannabis before midday, using cannabis alone, having memory problems when using cannabis, having friends or family telling them to reduce their cannabis use, experiencing problems such as arguments or fights as a result of cannabis use

Other substance use and dependencies at age 24

Participants reported any use in the past 12 months of the following: powder cocaine, crack cocaine, amphetamines, nitrous oxide, inhalants, sedatives, hallucinogens, opiates, or injected drugs. Reporting use of any one of these drugs was categorised as recent other illicit drug use.

Covariates

Prospective Measures from Early Childhood and Adolescence

Childhood socioeconomic position was assessed through measures from maternal questionnaires completed during pregnancy; variables were maternal educational attainment (university degree/A level or advanced level/O level, or less than O level, which includes any other qualifications of a lower academic standard or having no qualifications), and parents occupation class (i/ii/iii or iv/v).

© 2020 Hines LA et al. *JAMA Psychiatry*

Analysis

Missing data and imputation

As outcomes and exposures were collected at the same time point, the majority of missing data were in the covariates assessed at earlier ages (see Table 1). Missing data in all analysis variables (exposures, outcomes, covariates) were addressed through multiple imputation using chained equations, which uses a series of univariate regression models to impute each incomplete variable sequentially. Each model included all other analysis variables as predictors, along with the following auxiliary variables: experiencing bullying between ages 0-16, parental separation ages 0-16, parent mental health problems age 0-16, parent substance use age 0-16, MFQ score at age 16 and 18, number of self-reported psychotic-like experiences at age 14, and conduct disorder symptoms to age 13. Estimates were obtained by pooling results across 40 imputed datasets using Rubin's rules, and assessment of Monte Carlo variability confirmed this as a suitable number of imputations.[7]

© 2020 Hines LA et al. *JAMA Psychiatry*

eAppendix 2. Sensitivity Test Using Propensity Score Models in Complete Case Data

We have generated a propensity score in the complete-case data using logistic regression (outcome: reported cannabis potency) and the predict command in Stata 15.1. The variables and interactions included in the score are listed in eTable 1.

eTable 1. Variables and Interactions Included in Propensity Score

Individual variables	Interaction terms
Gender	PLIKS score age 12 x MFQ score age 12
Parent's socioeconomic status	Gender x MFQ score age 13
Maternal education	Gender x PLIKS score age 12
Frequency of cannabis use	Age of cannabis onset x MFQ score age 13
MFQ score at age 13	Age of cannabis onset x PLIKS score age 12
Number of psychotic-like experiences at age 12	Age of cannabis onset x gender
Age of cannabis onset	Age of cannabis onset x Parent's socioeconomic status
Class of conduct problems [8]	Parent's socioeconomic status x MFQ score age 13
Being bullied age 0-16 [9]	Parent's socioeconomic status x PLIKS score age 12
Parent substance problems when child age 0-16 [9]	Parent's socioeconomic status x gender
Caregiver mental health problems age 0-16 [9]	Parent's socioeconomic status x age of cannabis onset
Parental separation age 0-16 [9]	

This resulted in a model with R^2 of 0.26, and _hat predictor P Value of ≤0.001 (indicating good specification of the model). The score was applied to the data using Inverse Probability Weights (IPW). As IPW is sensitive to very large numbers, the IPWs were truncated to the 95% percentile (4.3).

A comparison of the effect estimates for multivariable regression and IPW PSM in the complete case data are provided in eTable 2.

© 2020 Hines LA et al. *JAMA Psychiatry*

eTable 2. Effect Sizes for Models of Relationship Between Cannabis Potency and Study Outcomes, Comparing Multivariable Adjustment and Inverse Probability Weighting by Propensity Score

Outcome	Multivariable adjustment* Odds Ratio (95% Confidence Intervals)	IPW propensity score Odds Ratio (95% Confidence Intervals
Cannabis problems	4.31[1] (1.28 – 14.47)	12.07 (4.49 – 32.43)
Other illicit drug use	1.12[1] (0.64 – 1.97)	2.13 (1.29 – 3.51)
Tobacco dependence	1.30[1] (0.55 – 3.09)	2.57 (1.30 – 5.09)
Alcohol Use Disorder	1.07[1] (0.56 – 2.03)	1.35 (0.77 – 2.39)
Depression	1.17[2] (0.51 – 2.70)	1.12 (0.61 – 2.02)
Generalised Anxiety Disorder	1.67[2] (0.84 – 3.32)	1.55 (0.93 – 2.57)
Psychotic-like experiences	2.78[3] (1.34 – 5.75)	1.63 (0.88 – 3.03)

*All multivariable models adjusted for gender, parent socioeconomic status, maternal education, frequency of cannabis use. Additional adjustment for [1]Age of cannabis onset [2]MFQ score at age 13 [3]Number of psychotic-like experiences at age 12

eTable 3. Sample Characteristics of 1087 Participants Who Reported Recent Cannabis Use (Numbers and Proportions From Complete-Case Data)

Variable	N	%	% missing
Use of high potency cannabis	139	12.8	6.6
Regular cannabis use	246	22.6	0
Recent cannabis use problems	22	2.0	0
Recent use of other illicit drugs	746	68.6	2.2
Recent tobacco dependence	195	17.9	0
Recent alcohol use disorder	115	10.6	5.1
Major Depression (moderate/severe symptoms)	108	9.9	0.1
Generalised Anxiety disorder	137	12.6	0.6
Psychotic-like experiences	84	7.7	3.5
Male	511	47.0	4.5
Low maternal education	151	13.9	9.1
Lower parent occupational class	321	29.5	13.2
Black or minority ethnic group	57	5.2	10.4%
	**Mean**	**Confidence intervals**	**% missing**
Age of cannabis use onset	16.7	16.5 – 16.9	0.1
MFQ score age 13	5.6	5.3 – 5.9	22.5
Number PE age 12	0.2	0.17 – 0.26	18.4

© 2020 Hines LA et al. *JAMA Psychiatry*

eTable 4. Prevalence of Demographic, Substance Abuse and Mental Health Outcomes in 2805 Participants Who Did Not Report Recent Cannabis Use (Numbers and Proportions From Complete-Case Data)

	**No recent cannabis use N=2805**	
	**N**	**%**
Lifetime cannabis use (prior to past year)	1271	45.31
Recent use of other illicit drugs	487	17.45
Recent tobacco dependence	135	4.81
Recent alcohol use disorder	48	1.81
Major Depression (moderate/severe symptoms)	186	6.66
Generalised Anxiety disorder	238	8.53
Psychotic-like experiences	142	5.21
Male	945	33.70
Low maternal education	462	18.38
Lower parent occupational class	505	38.06
Black or Minority Ethnic Group	91	3.67
	**Mean**	**Confidence Intervals**
Age of cannabis use onset	17.15	17.01 – 17.29
MFQ score age 13	4.81	4.64 – 5.00
Number PE age 12	0.01	0.14 – 0.19

© 2020 Hines LA et al. *JAMA Psychiatry*

eReferences.

1. Northstone K, Lewcock M, Groom A, et al. The Avon Longitudinal Study of Parents and Children (ALSPAC): an update on the enrolled sample of index children in 2019. *Wellcome Open Res*. 2019;4. doi:10.12688/wellcomeopenres.15132.1

2. Harris PA, Taylor R, Minor BL, et al. The REDCap consortium: Building an international community of software platform partners. *J Biomed Inform*. 2019;95:103208. doi:10.1016/j.jbi.2019.103208

3. Harris PA, Taylor R, Thielke R, Payne J, Gonzalez N, Conde JG. Research electronic data capture (REDCap)—A metadata-driven methodology and workflow process for providing translational research informatics support. *J Biomed Inform*. 2009;42(2):377-381. doi:10.1016/j.jbi.2008.08.010

4. Freeman TP, Morgan CJA, Hindocha C, Schafer G, Das RK, Curran HV. Just say "know": how do cannabinoid concentrations influence users' estimates of cannabis potency and the amount they roll in joints? *Addict Abingdon Engl*. 2014;109(10):1686-1694. doi:10.1111/add.12634

5. Forti MD, Quattrone D, Freeman TP, et al. The contribution of cannabis use to variation in the incidence of psychotic disorder across Europe (EU-GEI): a multicentre case-control study. *Lancet Psychiatry*. 2019;0(0). doi:10.1016/S2215-0366(19)30048-3

6. Legleye S, Karila L, Beck F, Reynaud M. Validation of the CAST, a general population Cannabis Abuse Screening Test. *J Subst Use*. 2007;12(4):233-242. doi:10.1080/14659890701476532

7. White IR, Royston P, Wood AM. Multiple imputation using chained equations: Issues and guidance for practice. *Stat Med*. 2011;30(4):377-399. doi:10.1002/sim.4067

8. Barker ED, Maughan B. Differentiating Early-Onset Persistent Versus Childhood-Limited Conduct Problem Youth. *Am J Psychiatry*. 2009;166(8):900-908. doi:10.1176/appi.ajp.2009.08121770

9. Houtepen LC, Heron J, Suderman MJ, Tilling K, Howe LD. Adverse childhood experiences in the children of the Avon Longitudinal Study of Parents and Children (ALSPAC). *Wellcome Open Res*. 2018;3:106. doi:10.12688/wellcomeopenres.14716.1
